# Quantitative proteomics of molybdenum cofactor biosynthesis and utilization in *Caenorhabditis elegans*

**DOI:** 10.1016/j.jbc.2026.113545

**Published:** 2026-09-08

**Authors:** Noah Ditz, Holger Eubel, Sushila Bhattacharya, Kurt Warnhoff, Kevin Oliphant, Ralf R. Mendel

**Affiliations:** 1 Institute of Plant Genetics, Leibniz University Hannover, Hannover, Germany; 2 Center for Genetics and Rare Diseases Research, Sanford Research, Sioux Falls, South Dakota, USA; 3 Department of Pediatrics, Sanford School of Medicine, University of South Dakota, Sioux Falls, South Dakota, USA; 4 Department of Biochemistry, University of Oxford, Oxford, United Kingdom; 5 Department of Plant Biology, Technical University Braunschweig, Braunschweig, Germany

**Keywords:** caenorhabditis elegans, label-free quantification, molybdenum cofactor, proteome stability, quantitative proteomics

## Abstract

The molybdenum cofactor (Moco) is a chemically labile prosthetic group required by a small but essential set of metazoan enzymes, including sulfite oxidase, xanthine dehydrogenase, aldehyde oxidases, and the mitochondrial amidoxime reducing components (MARC). Disruption of Moco biosynthesis in humans causes Molybdenum Cofactor Deficiency, a severe neonatal encephalopathy. *Caenorhabditis elegans* is unique among animals studied so far in that it can meet its Moco requirement through both endogenous biosynthesis and direct uptake of mature Moco from its bacterial diet. However, the organism-wide abundance of the Moco biosynthetic machinery and Moco-dependent enzymes, and their response to altered Moco supply, has remained unknown. Here, using data independent acquisition proteomics with histone anchored absolute quantification, we generated an organism wide quantitative atlas of Moco biosynthesis and utilization in *C. elegans* under standard and Moco limiting conditions. Components of the biosynthetic pathway showed a strikingly asymmetric abundance. The mitochondrial enzyme MOC-5, which catalyzes the first committed step in Moco biosynthesis, was present at only about 120 copies per genome equivalent, roughly fifty-fold below the downstream cytoplasmic biosynthetic machinery, which ranged from about 5000 to 8500 copies per genome equivalent, identifying MOC-5 as a stoichiometric bottleneck. On the utilization side, the MARC paralogs were the dominant Moco consumers, with MARC-1 exceeding 20,000 copies per genome equivalent. Loss of dietary or endogenous Moco selectively depleted the nonsulfurated clients SUOX-1 and MARC-1, whereas biosynthetic proteins remained unchanged, indicating that protein stability, rather than compensatory expression, are the main response to Moco limitation.

Molybdenum cofactor (Moco) is a chemically labile prosthetic group required for the activity of diverse enzymes across the tree of life (1). Structurally, Moco consists of a tricyclic pyranopterin scaffold that coordinates a single molybdenum atom and is synthesized by a conserved four-step biosynthetic pathway (2). In animals, four enzyme classes require Moco at their catalytic centers: sulfite oxidase (SUOX), which catalyzes the detoxification of sulfite to sulfate; xanthine dehydrogenase (XDH), a key enzyme in purine catabolism; aldehyde oxidases (AOX); and mitochondrial amidoxime reducing components (MARC), which are implicated in diverse detoxification reactions (3, 4).

Loss of Moco in humans causes Molybdenum Cofactor Deficiency, a devastating neonatal encephalopathy characterized by intractable seizures, sulfite toxicity, and profound neurological impairment (4). At present disease-modifying treatment is limited to early cyclic pyranopterin monophosphate substitution in Molybdenum Cofactor Deficiency type A (5, 6), whereas most patients lack an effective therapy. Isolated deficiencies of SUOX and XDH similarly give rise to severe metabolic disease (7, 8), and the mammalian mARC enzymes have emerged in the last decade as major catalysts of the reduction of N-hydroxylated drugs, prodrugs, and dietary xenobiotics (9). Despite this breadth of human-relevant biology, the absolute abundance and stoichiometry of the Moco biosynthetic and Moco-dependent proteins have not been resolved at an organism-wide level in any metazoan, leaving open basic questions about where flux through the pathway is set and which enzymes dominate its utilization *in vivo*. The Moco biosynthetic pathway and Moco-dependent enzymes present in the nematode *Caenorhabditis elegans* are summarized in Figure 1.

**Figure 1:**
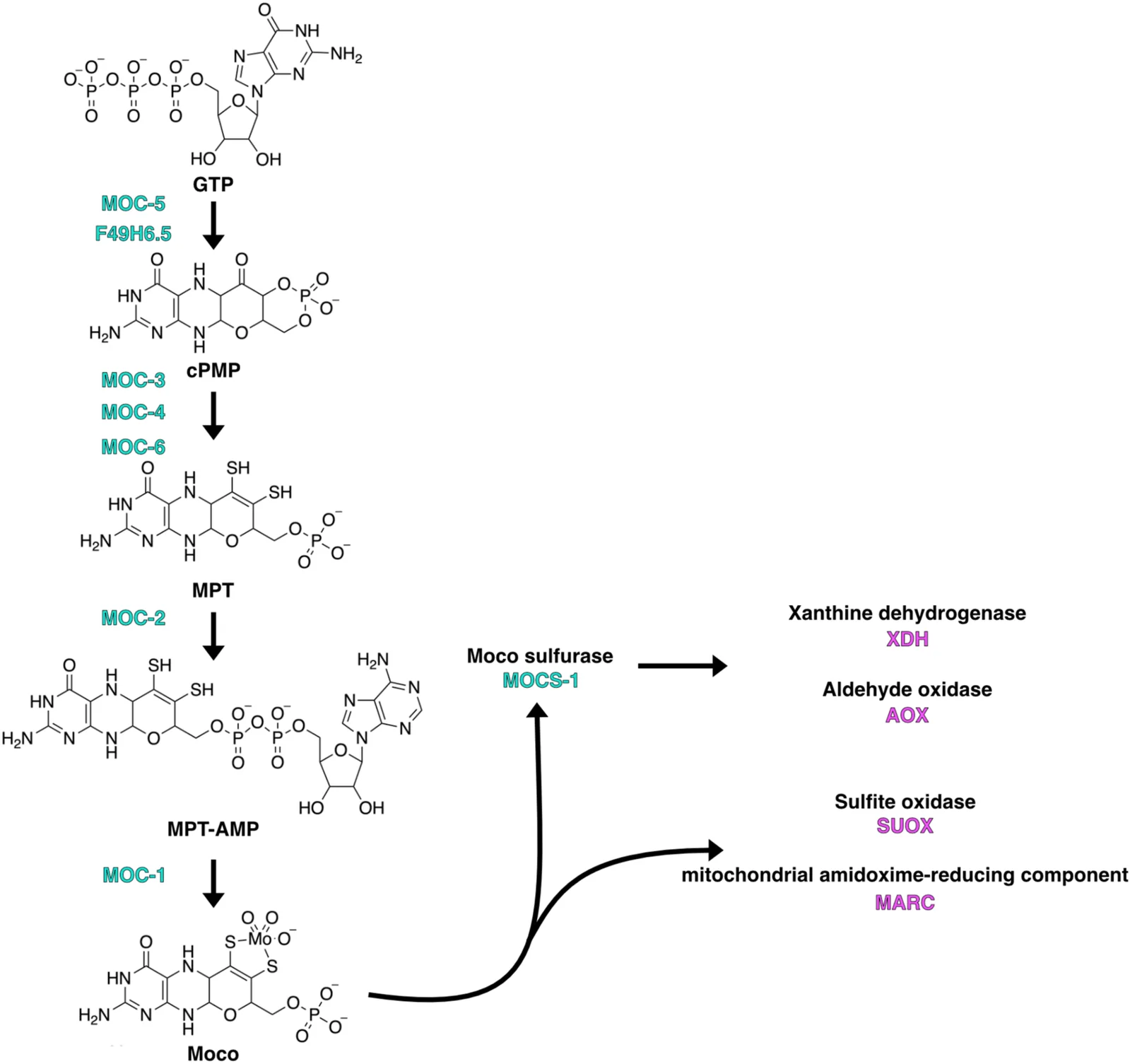
**Moco biosynthesis and Moco-dependent enzymes in *C. elegans*.** Structures of the Moco biosynthetic intermediates guanosine triphosphate, cyclic pyranopterin monophosphate, molybdopterin, molybdopterin adenosine monophosphate, and the mature Moco are shown. Enzymes involved in Moco biosynthesis (MOC-1 to MOC-6) are indicated in *green*. Following biosynthesis, Moco can be sulfurated by the putative Moco sulfurase MOCS-1, a modification required for insertion of Moco into xanthine dehydrogenase and aldehyde oxidase. Non-sulfurated Moco is inserted into sulfite oxidase and mitochondrial amidoxime reducing component. Moco, molybdenum cofactor Quantitative proteomics of molybdenum cofactor proteins.

In most organisms, Moco-requiring cells are thought to rely exclusively on *de novo* biosynthesis to satisfy their cofactor requirement. In *C. elegans*, Moco is essential for viability, as animals deficient in Moco arrest development at the larval stage and die. This lethality is attributable to the loss of SUOX activity, resulting in the accumulation of toxic sulfite (10, 11). SUOX-1 acts primarily in the *C. elegans* hypodermis, as hypodermal expression of *suox-1* is sufficient to rescue the larval lethality of *suox-1* null mutants (12, 13), underscoring the central importance of this enzyme for organismal survival. In mammals, Moco-dependent enzymes are expressed in tissues including the liver, kidney and brain, where they contribute to sulfite detoxification, purine metabolism and xenobiotic metabolism (14, 15, 16).

Unlike other metazoans studied to date, *C. elegans* exhibits a unique strategy for Moco homeostasis. In addition to endogenous biosynthesis, the nematode can acquire mature Moco from its bacterial diet and distribute it between tissues (10). Dietary Moco is stabilized by Moco-binding or Moco-dependent proteins and is transported by mechanisms that remain incompletely characterized, ultimately supporting the activity of Moco-dependent enzymes throughout the animal (12, 17). As a consequence, *C. elegans* carrying null mutations in genes encoding Moco biosynthetic enzymes remain viable and fertile when provided with dietary Moco. This exceptional biology has established *C. elegans* as a model system for studying Moco utilization and inter-tissue cofactor supply, with potential relevance for understanding Moco deficiency in higher organisms. Although inter-tissue trafficking of protein-bound Moco has been demonstrated in *C. elegans* (12), comparable intercellular transfer has not yet been demonstrated in mammals, and whether mature Moco is transported between mammalian cells remains unresolved.

Following ingestion, dietary Moco must be absorbed in the intestine and transported to distal tissues such as the hypodermis, where it supports SUOX-1 activity. Dietary and endogenously synthesized Moco contribute non-redundantly to total Moco availability and SUOX-1 function. Removal of either source results in decreased Moco content, decreased SUOX-1 activity, and increased sensitivity to sulfite, indicating that both pathways are required for Moco homeostasis (12, 17).

These observations raise the question of how Moco availability impacts Moco-dependent enzyme abundance. In humans, the loss of Moco leads to instability of SUOX (18). In *C. elegans,* the absence of dietary Moco reduces SUOX-1 enzymatic activity to approximately one third of that observed under standard conditions (17). Despite this reduction, animals relying solely on endogenous Moco biosynthesis develop normally but are more sensitive to elevated sulfite levels, whereas simultaneous loss of dietary and endogenous Moco results in larval arrest and lethality (10, 17). While the response of SUOX-1 activity to altered Moco supply has been characterized, it remains unknown how the abundance of Moco-dependent enzymes, as well as the Moco biosynthetic machinery itself, responds to changes in Moco availability.

Here, we use quantitative proteomics to determine the abundance of all Moco-dependent enzymes and the full set of Moco biosynthetic proteins in *C. elegans*. We further assess how protein abundance changes when dietary Moco is absent and when endogenous Moco biosynthesis is genetically disrupted using a *moc-1* loss-of-function mutant. Finally, we evaluate the extent to which altered Moco supply impacts the broader proteome beyond Moco metabolism.

## Results

### Identification of *C. elegans* protein groups by shotgun proteomics

To generate distinct Moco-limiting conditions, we cultured i) WT *C. elegans* on Moco-producing *Escherichia coli* (Moco-replete conditions), ii) WT *C. elegans* on Moco-deficient *E. coli* (inducing a lack of dietary Moco) or iii) *moc-1(ok366)* null mutant *C. elegans* on Moco-producing *E. coli* (inducing a lack of endogenous Moco). *moc-1* mutant animals cannot be cultured on a Moco-deficient diet as they undergo completely penetrant larval arrest followed by death (10). For proteomic analysis of each condition, proteins were extracted from approximately 15,000 young adult nematodes per biological replicate using SDS-based lysis, which were subsequently subjected to tryptic digestion. Of the resulting peptides, 200 ng were analyzed by LC-MS/MS operated in data-independent acquisition (DIA) mode for each sample.

Database searches against the *C. elegans* reference proteome (UniProt) resulted in the identification of approximately 7800 protein groups per sample (Table S1), corresponding to ∼40% of the predicted proteome. Relative protein abundance was determined using label-free quantification (LFQ) values calculated by the MaxLFQ algorithm (19). In addition, absolute protein abundance was estimated using intensity-based absolute quantification (iBAQ) values (20).

### Abundance of protein groups involved in Moco metabolism

iBAQ-based quantification allowed simultaneous assessment of proteins involved in Moco biosynthesis and utilization in *C. elegans* grown under standard laboratory conditions. Protein copy numbers were estimated using a modified proteomic ruler approach (21). Briefly, the summed iBAQ intensity of the detected histone (H2A, H2B, H3, H4) proteins and their variants was used to estimate the number of genome equivalents (GEs) present in each sample. Assuming a haploid genome size of ∼100 Mb (22) and ∼150 bp of DNA per nucleosome, each haploid GE corresponds to ∼666,667 nucleosomes and ∼5.3 million core histone molecules. Summed histone iBAQ intensities were divided by the calculated histone copy numbers for each GE to produce scaling factors transforming iBAQ values into estimated copy numbers for all other identified protein groups (Table S2). Because this normalization is anchored to total DNA content, it inherently accounts for all nuclei in the lysate, including diploid somatic cells, polyploid intestinal cells, and germline nuclei, without requiring assumptions about cell number or ploidy distribution.

The resulting scaling factors ranged from 0.85 to 1.22 between samples (Table S2). Because this GE-based normalization reflects total DNA content in the lysate, contributions from somatic, germline, and syncytial nuclei are inherently included. Given that *C. elegans* contains cells of varying ploidy—including diploid somatic cells, polyploid intestinal cells, and the syncytial germline—protein copy numbers per individual cell will differ from the per-GE values reported here, with diploid cells containing approximately twice the copies per GE. Reported values should therefore be considered estimates intended to facilitate comparisons of protein abundance rather than precise absolute quantification on a per-cell basis.

Fourteen Moco-related proteins were identified, ranging from approx. 120 to >20,000 copies per GE (Fig. 2 and Table 1). The cytoplasmic Moco biosynthesis enzymes (MOC-1, MOC-2, MOC-3, MOC-4 and MOC-6) were detected at comparable levels, typically between ∼5000 and ∼8500 copies per GE. In contrast, MOC-5, which catalyzes the first step of Moco biosynthesis in the mitochondria (10), was present at substantially lower abundance (∼120 copies per GE). Based on homology to characterized systems, MOC-5 is predicted to assemble a trimer of homodimers (23), which would correspond to approximately 15 to 20 hexameric complexes per GE, assuming complete assembly. MOC-5 is also predicted to function together with its paralog F49H6.5 (10), which was not detected. MOC-5 was identified by three peptides, of which one is unique to MOC-5; the remaining two are shared with its paralog F49H6.5. As no unique peptide was detected for F49H6.5, both proteins are combined within the same protein group, allowing neither a positive identification, nor a quantification of F49H6.5. The low number of peptides identified for MOC-5 likely reflects the low abundance of these proteins rather than limited proteomic sampling depth.

**Figure 2:**
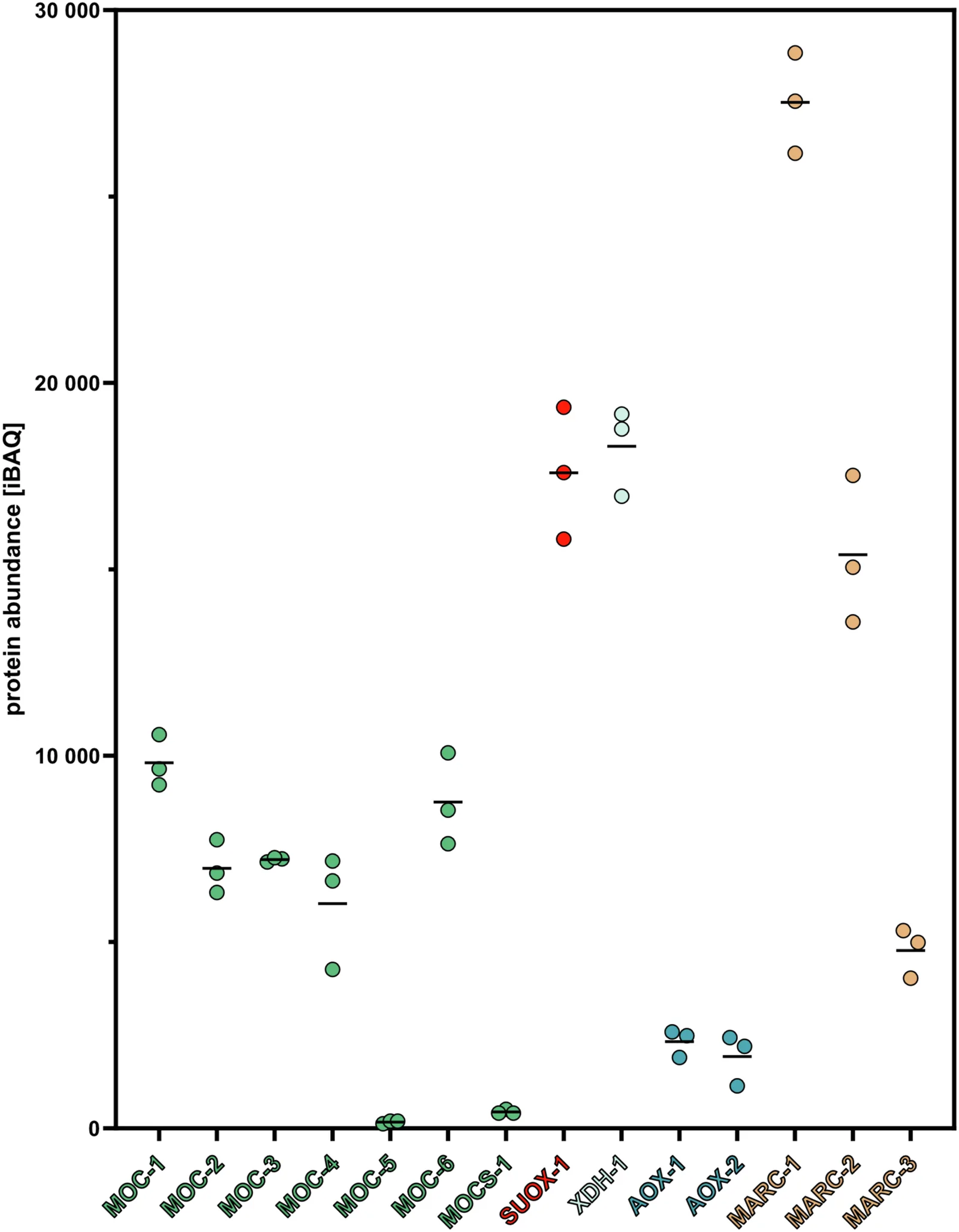
**Absolute abundance of Moco biosynthetic and Moco-dependent enzymes in *C. elegans*.** WT *C. elegans* were cultured on a diet of Moco-producing *Escherichia. coli*. Absolute protein abundance was estimated using iBAQ. The y-axis shows iBAQ values for the indicated protein groups derived from three biological replicates. *Horizontal bars* denote mean iBAQ values. Moco, molybdenum cofactor; iBAQ, intensity-based absolute quantification.

**Table 1:** Estimated copy numbers of Moco metabolism-related protein groups per *Caenorhabditis. elegans* GE from WT *C. elegans* cultivated on Moco-replete *Escherichia. coli*

Protein group	Protein	Mean copies/GE	Standard deviation
Q20624	MOC-5	119	20
Q21657	MOCS-1	561	460
O61198	aldehyde oxidases-2 (F15E6.6)	1205	285
Q960A1	aldehyde oxidases-1 (GAD-3)	1874	254
P91321	MARC-3 (F53E10.1)	3809	527
Q23069	MOC-2	4977	1312
O44510	MOC-3	5899	439
P91500	MOC-4	6388	1638
Q09412	MOC-6	6986	1210
G5EGH2	MOC-1	8519	1016
O45394	MARC-2 (F22B8.7)	13,735	2563
Q9XTQ8	SUOX-1	14,174	1530
O17892	xanthine dehydrogenase-1	17,495	2279
O44517	MARC-1 (F56A11.5)	23,006	1952

Protein copy numbers were estimated using a histone-based proteomic ruler approach. Values are sorted in ascending order of estimated copy number per GE. Further methodological details are provided in the text.

GE, genome equivalent; MARC, mitochondrial amidoxime reducing component.

The MPT synthase complex comprises MOC-4 and MOC-6, and forms an α_2_β_2_ heterotetramer in characterized systems (24). In our dataset, MOC-4 and MOC-6 were detected at 6388 ± 1638 and 6987 ± 1211 copies per GE, respectively, corresponding to a MOC-6:MOC-4 ratio of ∼1.1:1 and indicating near-equimolar abundance. MOC-3, which putatively provides the sulfur carrier required for reactivation of the complex (25), was present at comparable levels (5899 ± 439 copies per GE).

In bacteria, the final steps of Moco biosynthesis are mediated by two separate proteins: MogA, which forms trimers (26), and MoeA, which forms dimers (27). In higher eukaryotes, these enzymes are integrated within a single molybdenum insertase protein, such as gephyrin or Cnx1, in which the MogA-like (G) and MoeA-like (E) domains retain their characteristic oligomeric properties (28). In mammals, the E domain of gephyrin additionally serves scaffolding functions beyond Moco biosynthesis (29). In *C. elegans*, the corresponding activities are encoded by two separate proteins. MOC-2 (G/MogA-like) was detected at 4977 ± 1312 copies per GE, whereas MOC-1 (E/MoeA-like) was present at 8519 ± 1016 copies per GE, corresponding to a MOC-1:MOC-2 ratio of ∼1.7:1. The relative enrichment of the E domain-like component indicates that its abundance is not strictly determined by equimolar assembly with the G-domain-like component, potentially reflecting additional functional roles analogous to the scaffolding functions described for the E domain of mammalian gephyrin (29).

The putative Moco sulfurase MOCS-1 was detected at markedly lower abundance than the other Moco biosynthetic enzymes (561 ± 460 copies per GE). MOCS-1 catalyzes the sulfuration of Moco required for activation of XDH and AOX (3). Its comparatively low abundance is consistent with sulfuration functioning as a catalytic modification step rather than a bulk biosynthetic process.

Among Moco-dependent enzymes, SUOX-1 was detected at 14,174 ± 1530 copies per GE and XDH-1 at 17,495 ± 2279 copies per GE. In contrast, the predicted aldehyde oxidase ortholog GAD-3 and the related protein F15E6.6 were detected at lower levels (1874 ± 254 and 1205 ± 284 copies per GE, respectively). Sequence annotation in UniProt and WormBase indicates that F15E6.6 shares 34% sequence identity with XDH-1 and 82% sequence identity with GAD-3, consistent with its classification as an AOX-like paralogue. As *C. elegans* encodes a single XDH (30), whereas multiple AOX paralogues are common among metazoans (14), F15E6.6 likely represents a second AOX-related enzyme.

Within the MARC family, the MARC-1 orthologous F56A11.5 was the most abundant member (23,006 ± 1952 copies per GE), whereas the related MARC-like proteins F22B8.7 (MARC-2) and F53E10.1 (MARC-3) were detected at lower levels (13,735 ± 2563 and 3809 ± 527 copies per GE, respectively). Thus, the most abundant MARC paralogue is approximately six-fold more abundant than the least abundant one under standard laboratory conditions.

### Lack of external Moco supply leads to limited changes in the *C. elegans* proteome

To determine whether the absence of dietary Moco induces changes in the proteome, *C. elegans* were grown on an *E. coli moaA* mutant that is unable to synthesize Moco due to disruption of the first step of the pathway (17). Under these conditions, worms rely entirely on endogenous Moco biosynthesis. Because animals raised on *moaA* mutant *E. coli* develop normally and remain fertile (10), they can be used for quantitative proteomic comparison with animals fed Moco-replete *E. coli* to assess global or Moco-specific alterations in protein abundance.

A total of 7176 protein groups meet the criteria for statistical analysis. For differential abundance analysis, LFQ values are used rather than iBAQ-derived copy numbers, as LFQ intensities undergo sample-to-sample normalization that makes them better suited for statistical comparisons across conditions. Changes in protein abundance between animals grown on Moco-replete and Moco-deficient diets are visualized using a volcano plot, in which each protein group is represented by a single data point (Fig. 3). The housekeeping proteins actin-2 (ACT-2) and glyceraldehyde-3-phosphate dehydrogenase-1 (GPD-1) show no significant differences between dietary conditions and served as internal references. Notably, only 18 protein groups exhibit statistically significant changes in abundance in response to the absence of dietary Moco (Table 2 and Fig. 3), indicating that the global proteome remains remarkably stable under these conditions.

**Figure 3:**
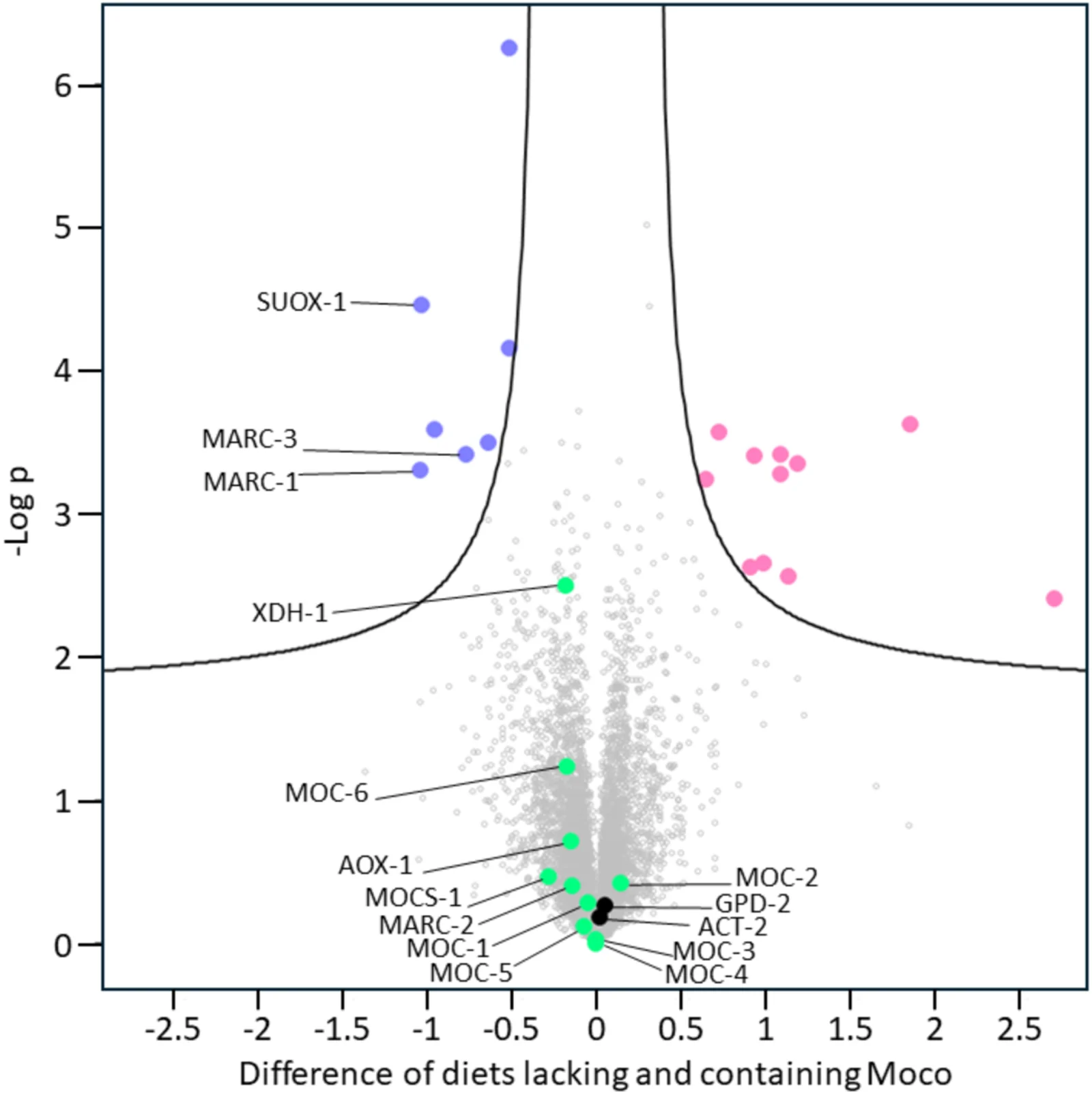
**Relative protein group abundance changes in *C. elegans* in response to dietary Moco availability.** Volcano plot of 7176 protein groups quantified in WT *C. elegans* grown on Moco-replete or Moco-deficient *E. coli*. The x-axis shows log_2_ fold changes in abundance (expressed as difference between Moco-deficient and Moco-replete), the y-axis displays -log_10_*p*-values from two-sample t-tests (n = 3 biological replicates; FDR 0.05; S0 = 0.1). Protein groups meeting significance criteria are highlighted: *blue*, significantly decreased under Moco-deficient conditions; *pink*, significantly increased; *green* Moco-related proteins not reaching significance (see Table 2). Housekeeping proteins actin-2 (ACT-2) and glyceraldehyde-3-phosphate dehydrogenase-1 are indicated in *black*. Moco, molybdenum cofactor.

**Table 2:** Differentially abundant protein groups in WT *C. elegans* in response to dietary Moco availability

Protein group	Genes	Description	*p* value (-log)	Difference
Q9XTQ8	*suox-1*	Sulfite oxidase	4.455273272	−1.036662102
Q966A6	Y58A7A.3	In between ring finger domain	3.58577461	−0.953680992
P91321	F53E10.1	MARC-3)	3.413490985	−0.771784782
H9G2U4;Q7JLY2	*dur-1*	Dauer up-regulated	3.495375198	−0.635720889
O45470	*oac-20*	Nose resistant-to-fluoxetine protein N-terminal DCP	6.252182656	−0.518404007
Q9U275	*haf-7*	Half ABC-transporter (P-glycoprotein related)	4.157414218	−0.511263212
O44517;U4PC34	F56A11.5	MARC-1	3.302929025	−1.04178524
Q9GYM9	*endu-2*	Endoribonuclease	3.350991547	1.19110616
Q21614;Q8WQD8	*cah-4*	Alpha-carbonic anhydrase DCP	3.243442738	0.649994214
P19974	*cyc-2.1*	Cytochrome c 2.1	3.56710149	0.727708181
G5ECD0	*cyp-35c1*	Cytochrome P450 family	2.625767833	0.907979329
O02628	*cyp-35a2*	Cytochrome P450 family	3.409062381	0.934602102
Q23605	ZK813.2	Neuropeptide-like protein	2.655163326	0.987623851
O18693	*acs-2*	Medium-chain acyl-CoA ligase ACSF2, mitochondrial	3.273510756	1.088029861
O76383	*hpo-15*	Amine oxidase DCP	3.415568437	1.088124593
Q9N5I1	*cyp-35a3*	Cytochrome P450 family	2.566409605	1.131827037
Q19661	F21C10.9	N-acetyltransferase DCP	3.626038976	1.852637609
Q8WSW2	C15B12.8	sarcosine oxidase (formaldehyde-forming)	2.409527941	2.703844388

Protein groups exhibiting significant abundance differences (FDR, 0.05; S0, 0.1) between wild-type *C. elegans* grown on Moco-replete or Moco-deficient *E. coli* are listed (see Fig. 3). Protein group, UniProt accession(s) corresponding to the identified proteins; Genes, gene names; Description, predicted or experimentally supported functional annotations; *p* value, two-sample Student’s *t* test result; Difference, mean difference in log2-transformed label-free quantification values. Negative difference values indicate reduced abundance in animals grown on Moco-deficient *E. coli*, whereas positive values indicate increased abundance under Moco-deficient conditions. DCP, domain-containing protein. Gene name colors match the scheme used in Figure 3.

MARC, mitochondrial amidoxime reducing component.

Proteins involved in Moco biosynthesis also remain largely unchanged upon removal of dietary Moco. In contrast, several Moco-dependent enzymes are significantly reduced in animals grown on Moco-deficient *E. coli*, including SUOX-1 and the putative MARC family members MARC-1 and MARC-3.

In addition to Moco-dependent enzymes, four additional protein groups are significantly reduced in animals lacking dietary Moco: the dauer-upregulated protein DUR-1, a predicted zinc-binding protein (Y58A7A.3), a half ABC transporter (HAF-7), and the transmembrane protein OAC-20. These proteins are not currently linked to Moco metabolism, and their decreased abundance may reflect secondary effects of dietary perturbation rather than direct involvement in cofactor homeostasis. Further genetic or functional analyses will be required to determine whether they play any role in Moco-related processes.

Proteins increased under Moco-deficient dietary conditions include multiple members of the cyp-35 family of cytochrome P450 enzymes (Table 2). In *C. elegans*, cyp-35 genes are well established as xenobiotic- and diet-responsive markers and are induced by exposure to diverse metabolic and environmental stressors (31, 32). Their upregulation here suggests that growth on Moco-deficient *E. coli* elicits a mild detoxification or metabolic stress response, rather than a specific compensatory activation of the Moco biosynthetic pathway. Among the other proteins increased under Moco-deficient conditions, ACS-2, a mitochondrial acyl-CoA synthetase that activates fatty acids as the first step of β-oxidation and is known to be induced by nutritional perturbation in *C. elegans*, was modestly elevated (33, 34), consistent with a broader dietary stress response rather than a direct consequence of altered Moco availability.

### Disruption of endogenous Moco biosynthesis selectively reduces Moco-dependent enzyme abundance

Having examined the effects of dietary Moco deprivation, we next assessed the impact of disrupting endogenous Moco biosynthesis. To this end, the proteome of *moc-1(ok366)* null mutants was compared with that of wild-type animals, with both strains being maintained on Moco-replete *E. coli*.

As in the dietary comparison, the housekeeping proteins ACT-2 and GPD-1 do not differ in abundance between genotypes (Fig. 4), indicating overall proteome stability. As expected, MOC-1 was not detected in the *moc-1* null mutant and is therefore not featured in the quantitative analysis.

**Figure 4:**
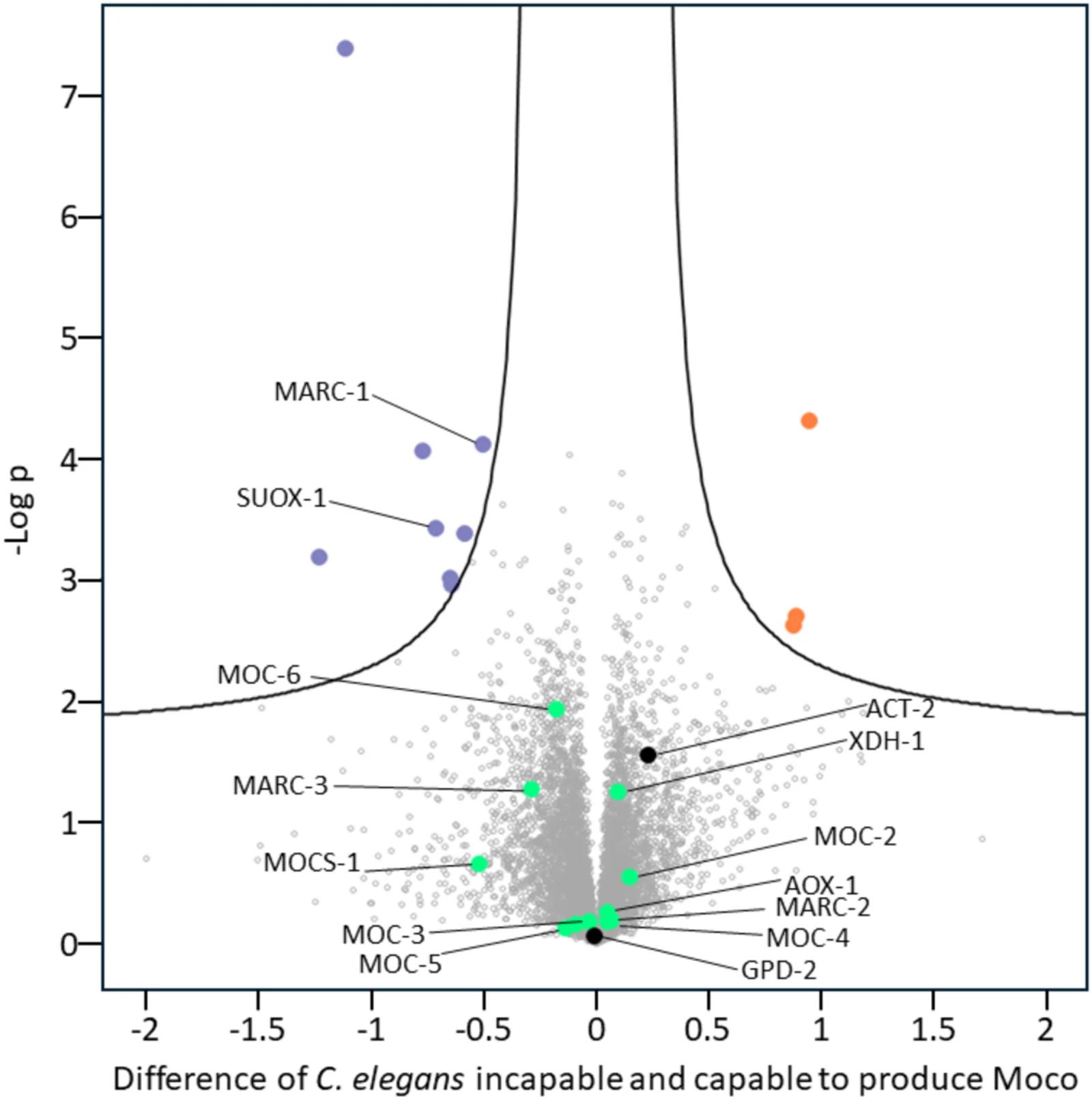
**Proteome-wide effects of loss of endogenous Moco biosynthesis in *C. elegans*.** Volcano plot of 7243 protein groups quantified in *moc-1* null mutants and WT animals, both grown on Moco-replete *E. coli*. The x-axis shows log_2_ fold changes between *moc-1* and WT, the y-axis displays -log_10_*p*-values from two-sample t-tests (n = 4 biological replicates for *moc-1* null mutants, n = 3 for WT; FDR 0.05; S0, 0.1). Significantly altered proteins are highlighted: *blue*, decreased in Δmoc-1; *orange*, increased in *Δmoc-1*; *green*, Moco-related proteins not reaching significance. Housekeeping proteins actin-2 (ACT-2) and glyceraldehyde-3-phosphate dehydrogenase-1 are indicated in *black*. Moco, molybdenum cofactor.

Overall, loss of endogenous Moco biosynthesis results in only limited proteomic changes. The abundance of other Moco biosynthetic enzymes remains unchanged in the mutant background. Similarly, most Moco-dependent enzymes do not show significant differences relative to wild-type. Notably, SUOX-1 and MARC-1 are significantly reduced in the *moc-1* mutant, consistent with the decreases observed under dietary Moco-deficient conditions.

In addition to the shared responses observed under both types of dietary Moco deprivation, 11 protein groups are significantly altered specifically in the *moc-1* mutant (Table 3), with eight of them decreasing and three increasing relative to wild-type. Aside from SUOX-1 and MARC-1, which are directly linked to Moco utilization, the affected proteins do not cluster within a defined metabolic pathway. Instead, several structural and cuticle-associated proteins are reduced, whereas innate immunity-related proteins such as LYS-7 and CLEC-66 are modestly increased. The observed changes are restricted to a small subset of structural and innate immunity-related proteins and do not extend to central metabolic pathways or the core Moco biosynthetic machinery. Comparison of the two datasets revealed limited overlap in differentially abundant proteins (Fig. 5). No proteins were significantly increased under both conditions, whereas SUOX-1 and MARC-1 were the only proteins significantly reduced in both datasets.

**Table 3:** Differentially abundant protein groups in *C. elegans* lacking endogenous Moco biosynthesis

Protein group	Genes	Description	*p*-value (-log)	Difference
Q20483	*gakh-1*	Protein Kinase	3.185803773	−1.229060253
Q20880	*col-121*	Nematode cuticle collagen N-terminal	4.068783793	−0.769828161
Q9XTQ8	*suox-1*	Sulfite oxidase	3.425729199	−0.713495811
H2KZ48	C30F12.5	Actin-binding anchor protein	3.018011908	−0.647911469
Q20713	F53B3.5	Conserved plasma membrane protein	2.95682153	−0.645014286
O44517;U4PC34	F56A11.5	Mitochondrial amidoxime reducing component (MARC-1)	4.118328257	−0.501051744
D0VWL6	*fln-2*	Calponin-homology	7.383844234	−1.11406374
O01571	F48C1.5	Homolog to photomorphogenesis genes	3.386612414	−0.58479015
O45445	*clec-66*	C-type lectin	2.627206849	0.874517123
Q9NAR3	*col-124*	Nematode cuticle collagen N-terminal	2.705359834	0.885226885
O16202	*lys-7*	Lysozyme-like protein 7	4.318467777	0.943823258

Only protein groups showing significant abundance differences (FDR, 0.05; S0, 0.1) between WT and *moc-1* mutant *C. elegans* are listed. Both strains were cultured on Moco-producing *E. coli* (see Fig. 4). Protein group, UniProt accession(s) corresponding to the identified proteins; Genes, gene names; Description, predicted or experimentally supported functional annotation(s); *p* value, two-sample Student’s *t* test result; Difference, mean difference in log2-transformed label-free quantification values. Negative values indicate lower protein abundance in the *moc-1 null* mutant, whereas positive values indicate higher protein abundance in the *moc-1 null* mutant. Gene name colors correspond to the color coding used in Figure 4.

**Figure 5:**
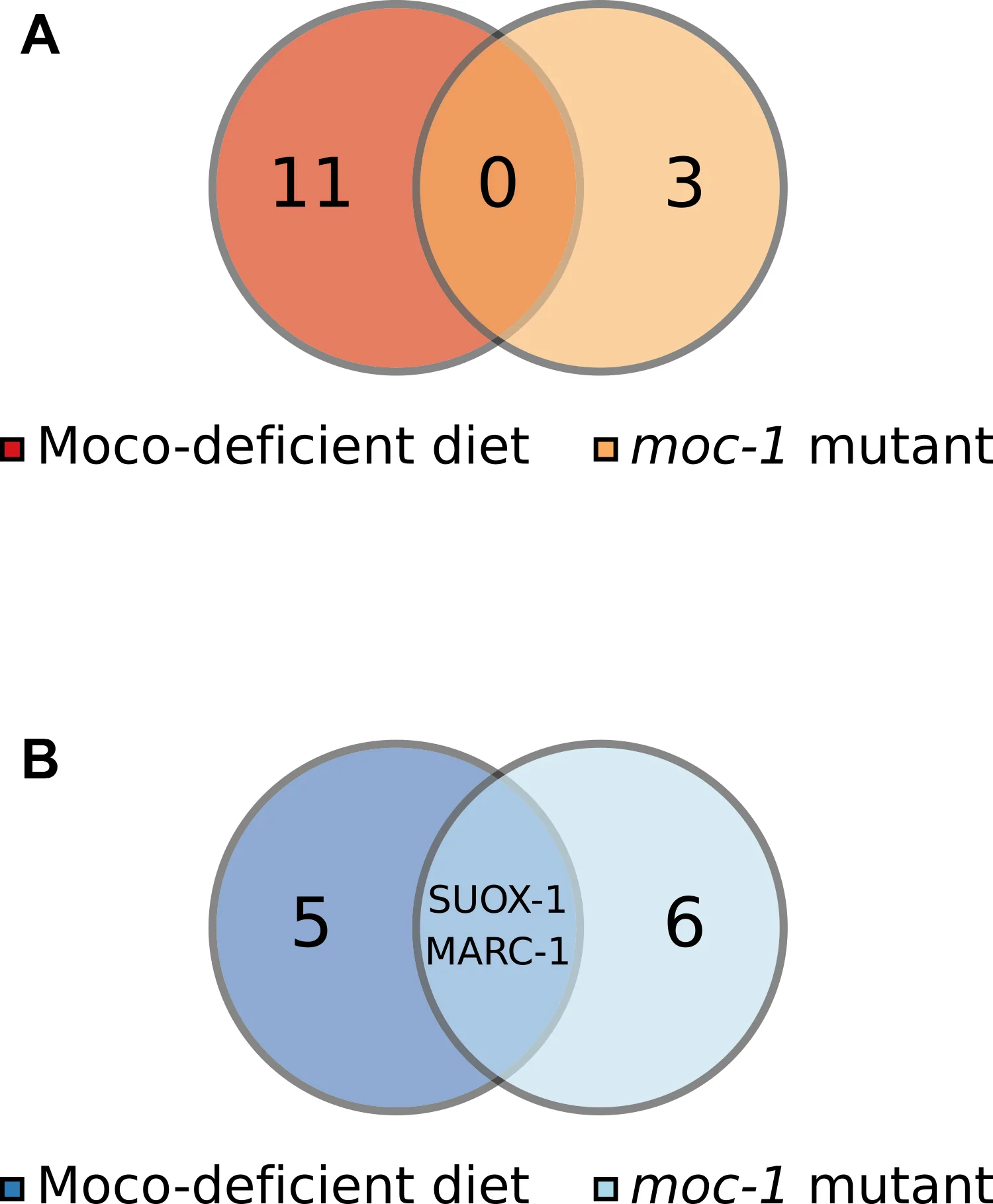
**Comparison of proteins differentially abundant in dietary Moco deficiency and disruption of endogenous Moco biosynthesis.***A*, overlap of proteins significantly increased under both conditions. *B*, overlap of proteins significantly decreased under both conditions. Moco, molybdenum cofactor.

Together, these proteomic analyses demonstrate that altered Moco availability results in a limited and reproducible set of protein abundance changes. Under standard laboratory conditions, neither loss of dietary Moco nor disruption of endogenous Moco biosynthesis induces broad proteome remodeling, likely due to their redundant roles in supporting development and reproduction (10). Apart from reductions in specific Moco-dependent enzymes, the overall proteomic landscape remains stable, consistent with the absence of overt physiological defects or phenotypes in these conditions.

## Discussion

In this study, we provide a quantitative inventory of enzymes involved in Moco biosynthesis and utilization in *C. elegans*. Across all conditions examined, proteins associated with Moco metabolism are present at substantially lower abundance than enzymes of central metabolic pathways. On average, tricarboxylic acid cycle enzymes are approximately 30 times more abundant than Moco biosynthetic enzymes (MOC-1 to MOC-4 and MOC-6) and ∼18 times more abundant than Moco-dependent enzymes, while enzymes involved in amino acid catabolism exceed Moco biosynthetic proteins by roughly factor 20 (Table S2). Comparison with other metal cofactor biosynthetic systems further illustrates the relative scarcity of Moco metabolism: core components of the mitochondrial iron-sulfur cluster assembly machinery (NFS-1, ISCU-1, FRH-1 and GLRX-5) are detected at abundance levels lower than central metabolic enzymes but higher than several Moco biosynthetic proteins (Table S2). These measurements place Moco metabolism among low-abundance but nevertheless essential enzymatic systems in the nematode.

Within the biosynthetic pathway, most enzymes were detected at similar abundance, with the notable exception of the mitochondrial MOC-5. MOC-5 catalyzes the first committed step of the pathway (10), converting GTP into the pterin precursor, and is present at markedly lower levels (∼100 copies per GE) than downstream components. Because this reaction consumes GTP-a central nucleotide required for protein synthesis and GTPase-mediated signaling (35) and which is also closely integrated with mitochondrial energy metabolism in *C. elegans* (36), the lower abundance of MOC-5 relative to the downstream cytoplasmic machinery identifies the mitochondrial, GTP-consuming entry into Moco biosynthesis as the stoichiometric bottleneck of the pathway. By holding MOC-5 at ∼100 copies per genome equivalent while maintaining the downstream machinery at ∼50-fold excess, the system decouples committed GTP consumption from the bulk capacity to process pathway intermediates, restricting entry into Moco biosynthesis to a tightly limited point. Maintaining a low abundance of MOC-5 may additionally minimize accumulation of the chemically labile intermediates generated during Moco biosynthesis, reducing the need to process unstable pathway metabolites.

Alternatively, the low abundance of MOC-5 may reflect adaptation to reduced demand under nutrient replete conditions. In this context cyclic pyranopterin monophosphate, known to be bioavailable to *C. elegans,* is readily supplied in the diet and does not need to be synthesized *via* MOC-5 (10). Notably, neither dietary Moco deprivation nor disruption of endogenous Moco biosynthesis produced a detectable increase in the steady-state abundance of the biosynthetic enzymes themselves, indicating that the pathway does not expand its biosynthetic capacity in response to Moco limitation at the protein abundance level.

The cytoplasmic biosynthetic enzymes (MOC-1, MOC-2, MOC-3, MOC-4 and MOC-6) are present within a relatively narrow abundance range (∼5000–8500 copies per GE). This stoichiometric similarity supports coordinated expression and is compatible with assembly into higher-order Moco biosynthetic complexes (37). Although our data do not directly demonstrate complex formation, the copy numbers are consistent with the proposed multi-enzyme organization of the pathway.

MOCS-1, which catalyzes sulfuration of Moco required for XDH and AOX activity but not for SUOX or MARC enzymes, was detected at lower abundance than the other biosynthetic components. Although three of the seven identified Moco-dependent enzyme species require sulfurated Moco, they represent only ∼27% of the total abundance of Moco-dependent proteins. The reduced steady-state abundance of MOCS-1 is therefore consistent with the limited demand for sulfurated cofactor.

Overall, the combined abundance of Moco-dependent enzymes (∼75,300 copies per GE) is approximately 2.2 times that of the biosynthetic machinery (∼33,000 copies per GE). This relationship indicates that cofactor biosynthesis operates at lower steady-state abundance than its client enzymes and supports a larger set of Moco-dependent proteins.

Despite the coordinated expression of the biosynthetic machinery, Moco-dependent enzymes showed selective responses to altered Moco availability. Enzymes utilizing the non-sulfurated form of Moco, including SUOX-1 and MARC-1, are reduced under both dietary Moco deprivation and disruption of endogenous biosynthesis. Notably, MARC-3 abundance is significantly reduced under dietary Moco limitation but not in the *moc-1* mutant, suggesting that MARC-3 may respond differently to the two types of Moco limitation, possibly because it is more sensitive to the absolute level of available dietary Moco than to reduced endogenous biosynthetic capacity. In contrast, enzymes requiring sulfurated Moco, such as XDH-1 and AOX family members, do not show significant changes in steady-state abundance.

SUOX-1 levels are reduced to approximately 50% of control following dietary Moco removal and to ∼60% upon loss of endogenous biosynthesis. This quantitative pattern parallels prior enzymatic measurements demonstrating that SUOX activity declines more strongly under dietary Moco deprivation than upon disruption of endogenous synthesis (17). Previous work in *C. elegans* showed that residual SUOX activity at these reduced levels is sufficient to sustain normal development but increases sensitivity to sulfite challenge (10, 17). In humans, partial SUOX activity is likewise compatible with viability, whereas complete loss-of-function results in severe disease (38, 39).

In contrast, XDH-1 abundance remains stable despite reduced Moco availability, indicating that Moco-dependent enzymes are not uniformly affected by cofactor limitation. In mammalian systems, incorporation of Moco is required for proper maturation and stability of SUOX, and impaired cofactor loading is associated with reduced protein stability and defective mitochondrial processing (18). A similar mechanism may operate in *C. elegans*, such that reduced Moco availability limits SUOX-1 stability or mitochondrial import rather than transcriptional regulation. XDH assembly, by contrast, involves distinct Moco insertion steps mediated by dedicated factors, and apoprotein forms can exist prior to cofactor incorporation without necessarily undergoing the stability constraints observed for SUOX (40).

The distribution of Moco-dependent enzymes also provides insight into how the cofactor may be allocated *in vivo*. XDH-1 is present at ∼17,500 copies per GE, whereas AOX enzymes were detected at substantially lower levels (∼1200–1900 copies per GE). Because aldehyde oxidases are widely implicated in xenobiotic metabolism, their comparatively low abundance likely reflects the benign laboratory growth conditions used in this study. Consequently, the values reported here probably represent basal expression levels rather than maximal physiological capacity. The MARC family member MARC-1 (F56A11.5) is the most abundant Moco-dependent enzyme (∼23,000 copies per GE), exceeding both XDH-1 and SUOX-1. Together, the three MARC paralogs account for a substantial fraction of the detected Moco-dependent proteome under the conditions examined. Within the purine degradation pathway, XDH functions downstream of purine nucleoside phosphorylase-1 and adenosine deaminase (ADA-2), and its abundance falls between the higher levels observed for purine nucleoside phosphorylase -1 (∼40,500 copies per GE) and the lower levels detected for ADA-2 (∼750 copies per GE) (Table S2). Although protein abundance alone does not directly reflect enzymatic flux or catalytic demand, the comparatively high abundance of MARC proteins suggests that MARC-dependent reactions constitute a major component of Moco utilization under standard laboratory conditions. The approximately six-fold range in abundance among the three MARC paralogues further suggests that these enzymes are unlikely to be functionally redundant. Although their endogenous substrates remain unknown in *C. elegans*, the marked predominance of MARC-1 is consistent with a constitutive metabolic function, whereas the lower abundance of MARC-2 and MARC-3 may reflect more specialized physiological roles or responsiveness to environmental conditions. Future studies examining tissue-specific expression and substrate preferences will be required to distinguish between these possibilities.

One limitation of the present study is that protein abundance was determined from whole-animal lysates. Consequently, tissue-specific changes in Moco metabolism may remain undetected if they occur in relatively small cell populations. Nevertheless, the reproducible reduction of SUOX-1, despite its primary functional role in the hypodermis, demonstrates that biologically meaningful responses can still be detected by whole-organism proteomics.

Together, these results define the quantitative landscape of Moco biosynthesis and utilization in an intact metazoan. The data reveal an asymmetry between largely invariant biosynthetic machinery and a subset of Moco-dependent enzymes whose abundance responds selectively to cofactor availability. These findings highlight protein abundance as an important organizational layer of Moco metabolism and provide a framework for understanding how fluctuations in cofactor supply can be accommodated without compromising organismal viability.

## Experimental procedures

### Culturing *C. elegans*

*C. elegans* strains were cultured using established protocols (41). Animals were grown at 20 °C on nematode growth media seeded with Moco-producing (WT, BW25113) or Moco-deficient (*ΔmoaA753::kan,* JW0764–2) *E. coli* (42). The WT *C. elegans* strain was Bristol N2. Strain GR2254 carrying the *moc-1(ok366)* deletion was used for studies of disrupted endogenous Moco synthesis (10).

For proteomic analyses, synchronized populations of ∼15,000 young adult hermaphrodite *C. elegans* were harvested per biological replicate. Synchronization was achieved by hypochlorite treatment of gravid adults to isolate eggs, followed by overnight arrest of hatched L1 larvae in M9 buffer without food (43), and subsequent transfer to nematode growth media plates seeded with Moco-producing or Moco-deficient *E. coli*. During sample collection, animals were washed extensively in M9 buffer to minimize contamination by ingested or surface-adherent bacterial cells. Despite these washing steps, a subset of bacterial proteins was detected in the proteomic datasets, consistent with residual bacterial material associated with the animals. To ensure accurate quantification of the nematode proteome, protein identification was performed against combined *C. elegans* and *E. coli* reference databases, and downstream analyses were restricted to protein groups uniquely assigned to the *C. elegans* proteome. Washed samples were then pelleted, supernatant removed, and freeze-dried (Harvest Right) prior to protein extraction and tryptic digestion.

### Protein extraction and tryptic digestion

Protein digestion was performed according to the single pot, solid phase enhanced sample preparation (SP3) protocol (44) with minor modifications. For this, worms were treated with 800 μl SDT buffer (4% [w/v] SDS; 0.1 M DTT; 0.1 M Tris-HCl, pH 7.6) and disrupted in a swing mill using four metal beads per tube at a frequency of 30 Hz for 5 min. After cell disruption, the SDT buffer was allowed to incubate for another 1 h in a shaker at 1000 rpm and 60 °C. Insolubilized material was pelleted at 20,000*g* for 10 min at room temperature. The supernatant was transferred to a new tube and incubated for 10 min in an ultrasonication bath, followed by centrifugation at 20,000*g* for 10 min at room temperature. The supernatant was used for protein quantification using the Pierce detergent compatible Bradford assay kit (Thermo Fisher Scientific) according to the manufacturer’s instructions. Protein concentration was adjusted to 2.5 μg μL^−1^ using SDT buffer, and 30 μl of the solution were transferred to a new tube. Cysteine residues were alkylated by addition of 7.5 μl 0.1 M iodoacetamide and incubation for 30 min at 600 rpm and 24 °C in a shaker. The reaction was stopped by addition of 2 μl 0.1 M DTT. A 1:1 mixture of hydrophilic and hydrophobic Sera-Mag Carboxylate-Modified Beads (GE Life Science) was freshly prepared and the sample was treated with 30 μl bead mixture (equivalent to 600 μg beads). The bead-protein mixture was supplemented with 70 μl 100% ethanol, followed by a 10 min incubation at 24 °C and 1000 rpm (shaker). Afterwards, beads were pelleted for 2 min in a magnetic rack. The supernatant was removed and beads were washed by resuspending them in 140 μl 80% [v/v] ethanol. Washing was repeated for three further times, before the sample was transferred into a new low-bind tube. Beads were pelleted for 2 min in a magnetic rack and the supernatant was removed. Beads were resuspended in 20 μl trypsin solution (0.1 μg μL^−1^ sequencing grade modified trypsin (Promega) in 50 mM acetic acid) that was pre-activated according to the manufacturer’s instructions. Afterwards, the solution was diluted with 40 μl ammonium bicarbonate, yielding a final concentration of 50 mM. Tryptic digestion was allowed to commence for 18 h at 37 °C and 1000 rpm (shaker). Peptides were subsequently separated by pelleting the beads for 2 min in a magnetic rack. The supernatant was transferred to new low-bind tubes and beads were resuspended in 60 μl 50 mM ammonium bicarbonate. Beads were again pelleted and supernatants were pooled with supernatants of the previous step. Tryptic activity was quenched by addition of 1.2 μl formic acid and peptides were desalted on Sep-Pak 50 mg C18 cartridges (Waters) according to the manufacturer’s instructions. Desalted peptides were dried in a vacuum centrifuge and resuspended in 100 μl 0.1% [v/v] formic acid for peptide quantification using the Pierce Quantitative Colorimetric Peptide Assay (Thermo Fisher Scientific) according to the manufacturer’s instructions. For subsequent analysis, samples were adjusted to peptide concentrations of 200 ng μL^−1^ using 0.1% [v/v] formic acid.

### Liquid chromatography coupled tandem mass spectrometry and data processing

Samples were transferred into glass vials and stored at 4 °C in the autosampler of a nanoElute2 nano-flow liquid chromatography device that was coupled to a timsTOF Pro mass spectrometer (both Bruker). One microliter of sample was injected into a 20 μl sample loop and directly loaded onto a 25 cm Aurora Ultimate CSI column with an inner diameter of 75 μm, 120 Å pore size, and a particle size of 1.7 μm (IonOpticks). Peptides were eluted using a 60 min 2% [v/v] to 37% [v/v] acetonitrile gradient in 0.1% [v/v] formic acid and ionized using a captive spray ion source using a temperature of 180 °C and a capillary voltage of 1600 V. The mass spectrometer was operated in a positive polarity mode using the pre-installed ‘dia-PASEF–long gradient’ method.

A spectral library was predicted based on the *C. elegans* reference proteome obtained from uniprot (Proteome ID UP000001940; date 15.01.2026) using the DIA-NN software version 1.9.2 (45) and the following settings: ‘Trypsin/P’ was selected as protease with a maximum of one missed cleavage allowed. Methionine excision of the N-terminus, carbamidomethylation of cysteine, oxidation of methionine, and acetylation of N-termini were enabled. Maximum number of variable modifications was set to 1. Peptides with a length between 7 to 30 and a precursor charge between 1 to 5 were considered. Precursor m/z range was set to 300 to 1700 m/z, fragment ion m/z was set to 200 to 1700 m/z. Recorded spectra were queried against the *in silico* spectral library using a machine learning with double-pass neural networks and mass accuracies of 15 ppm at a precursor FDR of 1%.

To obtain iBAQ (20) values, the DIA-NN output was analyzed using the DIAgui R-package (46). Peptide lengths were set to 7 to 30, trypsin was selected as a protease, and the MaxLFQ (19) algorithm from the DIA-NN package was used.

Statistical analysis was performed with the Perseus software version 2.1.2.0 (47). For each comparison, LFQ values were log2 transformed and filtered for protein groups with at least three values in each of the compared groups. Missing values were replaced from normal distribution for the comparison between WT and *moc-1 C. elegans.* To detect significant protein abundance differences, a *t* test was performed at a FDR of 0.05 and a S0 of 0.1. The number of randomizations was set to 250.

## Data availability

The mass spectrometry proteomics data generated in this study have been deposited in the ProteomeXchange Consortium *via* the MassIVE repository with the dataset identifier MSV000101433. Username for the reviewer web access: MSV000101433_reviewer with the password: WorMS.

Alternatively use the following link: ftp://MSV000101433@massive-ftp.ucsd.edu with the username: MSV000101433 and the password: WorMS.

Data will be publicly available upon publication.

## Supporting information

This article contains supporting information.

## Conflict of interest

The authors declare that they have no conflicts of interest with the contents of this article.

## Supporting information

Supplemetary.file
