# Supplementary material for: Quantitative proteomics of molybdenum cofactor biosynthesis and utilization in Caenorhabditis elegans
Source: J Biol Chem. 2026 Sep 8;302(10):113545. doi: 10.1016/j.jbc.2026.113545 (PMC13653925; doi:10.1016/j.jbc.2026.113545)
Supplement: Supplementary file 1 [file mmc1.docx]

**Supplementary File**

**Quantitative proteomics of molybdenum cofactor biosynthesis and utilization in *Caenorhabditis elegans***

**Noah Ditz^1^, Holger Eubel^1^, Sushila Bhattacharya^2^, Kurt Warnhoff^2,5^, Kevin Oliphant^3^* and Ralf R. Mendel^4^***

**^1^**Institute of Plant Genetics, Leibniz University Hannover, Hannover, D-30419, Germany.

^2^Center for Genetics and Rare Diseases Research, Sanford Research, Sioux Falls, SD 57104, USA.

^3^Department of Biochemistry, University of Oxford, Oxford, OX1 3QU, United Kingdom.

^4^Department of Plant Biology, Technical University Braunschweig, Braunschweig, D-38106, Germany.

^5^Department of Pediatrics, Sanford School of Medicine, University of South Dakota, Sioux Falls, SD 57105 USA.

*Correspondence: r.mendel@tu-braunschweig.de; kevin.oliphant@bioch.ox.ac.uk

**Keywords:** molybdenum cofactor, Caenorhabditis elegans, quantitative proteomics, label-free quantification, proteome stability

**Table S1. Quantitative proteomic dataset of *C. elegans* samples used for genome-equivalent–normalized protein abundance calculations.** Protein groups identified by DIA mass spectrometry together with corresponding gene annotations, protein descriptions, molecular masses, and iBAQ intensities for all biological replicates. The dataset comprises all proteins detected and quantified in the study and serves as the basis for subsequent genome-equivalent (GE) normalization and copy number estimation.

**Table S2. Calculation of genome-equivalent scaling factors and estimated protein copy numbers.** Workbook containing the complete calculations used to convert iBAQ intensities into estimated protein copy numbers per genome equivalent (GE) using a modified proteomic ruler approach. The workbook includes the biological constants used for GE estimation, histone-based calibration and scaling factor calculations for each sample, calculated copy numbers for Moco-related proteins, and copy number estimates for selected reference proteins. All calculations are provided to enable full transparency and reproducibility of the normalization procedure.
